# Metastatic Invasive Lobular Breast Cancer Presenting Clinically with Esophageal Dysphagia

**DOI:** 10.1155/2017/7065674

**Published:** 2017-01-16

**Authors:** Lilit Karapetyan, Heather Laird-Fick, Reuben Cuison

**Affiliations:** ^1^Department of Medicine, Michigan State University College of Human Medicine, 788 Service Road Suite B301, East Lansing, MI, USA; ^2^Pathology Department, EW Sparrow Hospital, 1215 East Michigan Avenue, Lansing, MI 48909-7980, USA

## Abstract

*Background*. Intra-abdominal metastases of invasive lobular breast cancer (ILBC) may be insidious. We report a case of metastatic ILBC that presented with dysphagia within weeks of a negative mammogram and before the development of intra-abdominal symptoms.* Case*. A 70-year-old female developed esophageal dysphagia. She underwent EGD which showed a short segment of stricture of the distal esophagus without significant mucosal changes. Biopsy was unremarkable and patient underwent lower esophageal sphincter (LES) dilation. Severe progressive dysphagia led to esophageal impaction and three LES dilatations. CT scan showed bilateral pleural effusions, more prominent on right side, and ascites. The pleural effusions were transudative. Repeat EGD with biopsy showed lymphocytic esophagitis, and she was started on swallowed fluticasone. Abdominal ultrasound with Doppler showed that the main portal vein had atypical turbulent flow that was felt to possibly be due to retroperitoneal process. The patient underwent diagnostic laparoscopy which revealed diffuse punctate lesions on the peritoneum. Pathology was consistent with metastatic ILBC.* Conclusion*. Dysphagia in the setting of peritoneal carcinomatosis from metastatic ILBC is a rare finding. The case highlights the importance of metastatic ILBC as a differential diagnosis for female patients with progressive dysphagia and associated ascites or pleural effusions.

## 1. Introduction

Breast cancer comprises 14.6% of total new cancer cases in the United States. Invasive lobular breast cancer (ILBC) is the second most common histologic type of breast cancer and accounts for 10–14% of all breast cancer cases [1]. The incidence of ILBC has increased in the US over time, particularly among postmenopausal women. An estimated 3–10% of women with ILBC present with metastatic disease. As with invasive ductal breast cancer (IDBC), metastases are most often to bone, lung, liver, and brain. Unlike IDBC, however, ILBC has a predilection for metastasis to the abdomen, peritoneum, and leptomeninges. Intra-abdominal metastases may be insidious and difficult to differentiate histologically from gastric adenocarcinoma [2]. We report a case of metastatic ILBC that presented with esophageal dysphagia within weeks of a negative mammogram and before the development of intra-abdominal symptoms.

## 2. Case Presentation

A 70-year-old female with hypertension, psoriatic arthritis, and up to date breast and colon cancer screening developed difficulty swallowing, characterized by food “sticking” in her distal esophagus, while in Arizona for the winter season, she underwent initial evaluation there. Esophagoduodenoscopy (EGD) showed distal esophageal stricture and small erosions; biopsy was nonspecific. The CT scan showed moderate to marked bilateral hydronephrosis to the UPJ level without obstructing calculi, scattered colonic diverticulosis and bilateral pleural effusions right greater than left. She was started on a proton pump inhibitor for possible reflux-related symptoms without improvement. She subsequently elected to return home for additional evaluation. She underwent repeat EGD which showed a short segment of stricture of the distal esophagus and gastroesophageal junction (lumen 2 to 3 mm in diameter) without significant mucosal changes. Biopsy was unremarkable and patient underwent LES dilation. Due to persistent food impactions in the distal esophagus, EGD was repeated and showed distal esophageal stricture, small erosions, and mild gastropathy. The patient had a esophagogram which showed no ulceration, mass or constrictive lesion involving the esophagus, and was not suggestive of achalasia. Repeat abdominal CT scan was unremarkable except stable bilateral hydronephrosis and small bilateral pleural effusions, larger on the right side. She was evaluated by urology, who could find no specific cause of hydronephrosis and felt it was most likely congenital.

**Figure 1:**
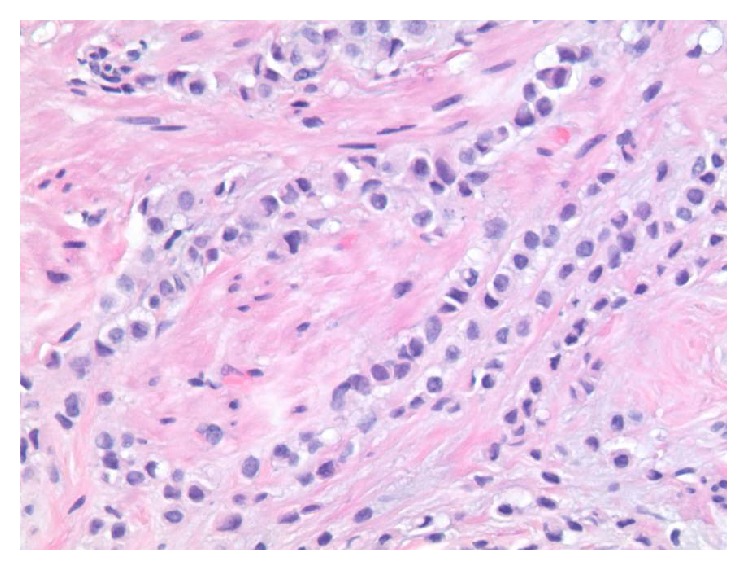
H&E stained slide demonstrating stromal infiltration by tumor cells arranged singly, in small clusters, and in single file pattern.

**Figure 2:**
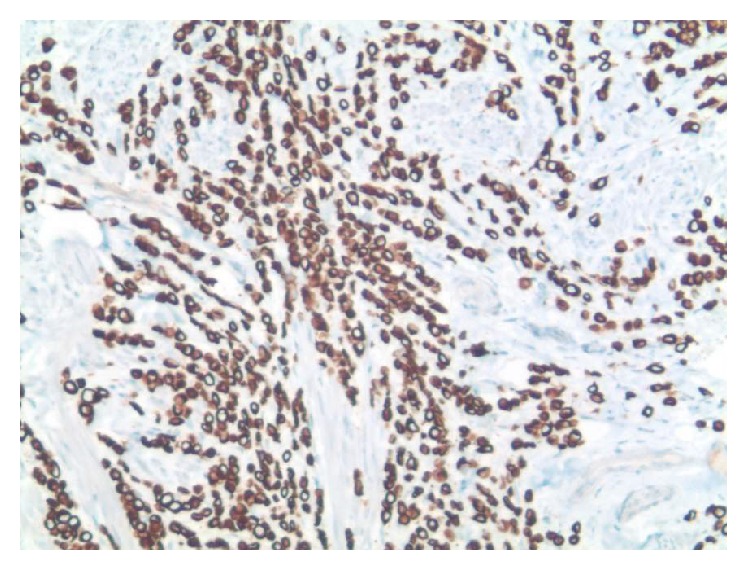
Positive immunohistochemical staining for CK7.

**Figure 3:**
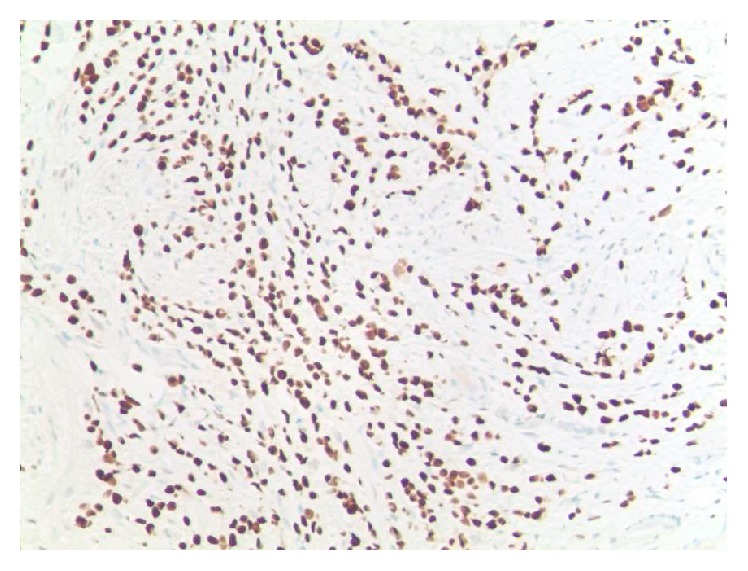
Positive immunohistochemical staining for ER.

The patient continued to slowly deteriorate. Severe progressive esophageal dysphagia led to hospitalization and subsequent esophageal disimpaction and three LES dilatations. Repeat CT scan showed worsening bilateral pleural effusions, most prominent on right side, and ascites. The pleural effusions were transudative, and necessitated six-large volume thoracentesis over four months due to shortness of breath. No paracentesis could be performed due to lack of distinct pocket. Due to history of psoriatic arthritis, autoimmune workup was done including antibodies for anti-smooth muscle, nuclear antigens, liver/kidney microsome type 1, alpha-1-antitrypsin, endomysial, and anti-mitochondrial but failed to explain dysphagia. Liver architecture, transaminases, alkaline phosphatase, and prothrombin time were normal. Because of ongoing progressive dysphagia and weight loss the patient was referred to a quaternary care center. The repeat EGD with biopsy showed lymphocytic infiltrates in the distal esophagus. She was diagnosed with lymphocytic esophagitis, started on swallowed fluticasone for esophagitis and furosemide for ascites and an appointment scheduled as an outpatient with a hepatologist for probable noncirrhotic portal hypertension. Additional bloodwork for causes of noncirrhotic portal hypertension was negative except elevated D-dimer of 5.52 mg/L. Abdominal ultrasound with Doppler showed that the main portal vein at the porta hepatis was patent but had atypical turbulent flow that was felt to possibly be due to proximal stenosis at the level of the splenic vein-portal confluence or a retroperitoneal process. Review of prior CT scans with interventional radiology and general surgery was not definitive for retroperitoneal infiltration of soft tissue. The patient underwent diagnostic laparoscopy which revealed diffuse punctate lesions on the peritoneum and liver and omental thickening. No discrete mass was identified. Ascitic fluid of 1800 mL was removed. Peritoneal, liver, and omental biopsies were obtained. Pathology was consistent with metastatic lobular breast carcinoma (Figure 1). Immunoperoxidase stains performed and showed diffuse and strong tumor nuclear staining with estrogen receptor (ER) and strong and diffuse tumor cytoplasmic staining with CK7 (Figures 2 and 3). Rare tumor nuclei stained with progesterone receptor while no reactivity was demonstrated with Her2 or CK20. The esophageal biopsy slides from the quaternary care center were obtained and reviewed by the surgical pathologist who interpreted the peritoneal biopsies. Benign reactive changes with intraepithelial lymphocytosis were found; the lymphocytes were histologically distinct from the cells seen on the peritoneal biopsies and fluid removed during surgery.

## 3. Discussion

ILBC differs in important ways from IDBC. ILBC has a different genomic profile compared with IDBC. ILBCs are characterized by 13q and 22q losses, resulting in loss of KLF5 which promotes cell proliferation. Biologically, most ILBCs lack expression of E-cadherin protein, which is responsible for cell adhesion and tissue binding [3]. ILBC is also characterized by decreased expression of cell adhesion molecule ADAM12, minimal desmoplastic reaction, and decreased inflammatory response of surrounding tissue [3].

These characteristics may explain the different metastatic pattern of ILBCs. Clinically, it is more likely to present with bilateral or multicentric involvement, at an advanced stage, a lower histological grade, increased lymph node positivity, and less vascular invasion. The ILBCs tend to metastasize to the gastrointestinal system (4.5% versus 0.2%), female reproductive organs (4.5% versus 0.8%), peritoneum and retroperitoneum (3.1 versus 0.6%), adrenal glands (0.6 versus 0%), bone marrow (21.2% versus 14.4%), bone (50% versus 34%), and lung pleura (2.5% versus 10.2%) [4, 5].

The overall recurrence rate, proportion of local recurrence rate, and proportion of distant recurrences were found to be similar in ILBC and IDBC [6, 7]. Due to low proliferative index and an indolent course, ILBCs respond less well to chemotherapy than do IDBCs.

Approximately 95% of cases are usually ER/PR positive, with less than 5% HER2 positivity. In 55–100% of cases ILBC lacks expression of E-cadherin protein. The loss of expression of the E-cadherin in infiltrating lobular carcinoma results in the cell spreading and dissemination. The cancer infiltrates the breast stroma in a single file, encircling mammary ducts and lobules. This pattern of spread results in normal anatomic structure of breast, without mass lesions on physical examination and imaging studies. Mammography has a lower sensitivity for detecting ILBC similar to that happened in our patient. Metastatic ILBC infiltrates to the target organs in a diffuse process again without forming discrete tumor nodules. CT scan usually shows numerous tiny nodules infiltrating the peritoneum and diffuse infiltration of solid organ walls instead of extramural masses. Immunohistochemical staining of breast metastases is usually positive for CK7 and GCDFP-15 and negative for CK20 [8].

Gastrointestinal tract metastases are rare and present in less than 1% of patients in clinical settings although they have been observed in 20–43% on autopsy series. Patients usually present with common symptoms like nausea and vomiting, diarrhea, and abdominal pain. This nonspecific clinical presentation can result in delayed diagnosis and treatment of metastatic disease [9].

Dysphagia is a rare initial presentation of metastatic ILBC. Metastases to the esophagus usually occur via lymphangitic spread and present as submucosal lesions with normal mucosa, thus resulting in normal EGD and esophagography [10, 11]. In a series of 24 cases, 20 presented with stricture, three patients with achalasia, and one patient with nonspecific dysmotility. Although endoscopy is the best diagnostic method for detecting upper GI pathologies, endoscopic biopsies are often falsely negative especially when they are superficial. In these cases, endoscopic ultrasound (EUS) with fine needle aspiration is helpful [12, 13]. However, our patient did not have EUS.

Patients with metastatic breast cancer have poor prognosis due to late presentation and advanced disease. The median survival time is 28 months. Our patient started treatment with paclitaxel which resulted in improvement of her dysphagia. In some cases peritoneal carcinomatosis can be treated with cytoreductive surgery and hyperthermic intraperitoneal chemotherapy. However, our patient was not a candidate for that due to widespread peritoneal metastases, diffuse omental thickening, and failure to demonstrate a discrete mass lesion [14].

Esophageal dysphagia in the setting of peritoneal carcinomatosis from metastatic ILBC is a rare finding. In our patient dysphagia might have been caused either by peritoneal carcinomatosis or by esophageal metastases, although the latter was not confirmed by the available pathologic specimens. As metastatic ILBC cells can easily be mistaken for lymphocytes, the surgical pathologist who interpreted the intra-abdominal biopsies also reinterpreted the esophageal biopsies from the quaternary care center. He felt strongly that the esophageal lymphocytes were typical and histologically dissimilar to the metastatic cancer cells. He declined additional staining. No additional surgical specimens of the esophagus were obtained.

The case highlights metastatic ILBC as a potential cause of progressive esophageal dysphagia in women. While fewer than 5% of patients in a recent Swedish study had esophageal food bolus impaction as a result of cancer [15], metastatic ILBC should be considered in those women with alarm symptoms (i.e., significant weight loss) who have had otherwise unrevealing work up or have additional unexplained findings such as ascites and pleural effusions. Although ILBC-induced dysphagia is rare, delay in diagnosis and therefore treatment affects quality of life.
